# Altered retinal nerve fiber layer thickness in children with allergic conjunctivitis: the Nanjing eye study

**DOI:** 10.1186/s12886-022-02399-7

**Published:** 2022-04-22

**Authors:** Danni Chen, Rui Li, Dan Huang, Haohai Tong, Xiaoyan Zhao, Wen Yan, Shiya Shen, Hu Liu, Hui Zhu

**Affiliations:** 1 Department of Ophthalmology, The First Affiliated Hospital with Nanjing Medical University, Nanjing, China; 2 The Second Affiliated Hospital, Zhejiang University School of Medicine, Eye Center, Hangzhou, Zhejiang China; 3 Department of Ophthalmology, The Affiliated Changzhou No.2 People’s Hospital of Nanjing Medical University, Changzhou, 213164 Jiangsu Province P.R. China; 4 Department of Ophthalmology, The Fourth Affiliated Hospital of Nantong University, The First people’s Hospital of Yancheng, Yancheng, China

**Keywords:** Allergic conjunctivitis, Retinal thickness, Vessel density, School children, Optical

## Abstract

**Background:**

So far, few data are available on the relationship between allergic conjunctivitis (AC) and ocular fundus. Whether retinal parameters change in patients with AC remains unknown. In this study, we investigated the influence of AC on retinal thickness and vessel density among 7-years-old school-age children.

**Methods:**

This large population-based study is part of the Nanjing Eye Study (NES). Comprehensive examinations including anthropometric parameters, refraction, ocular biometric parameters, intraocular pressure and retinal parameters were conducted on each child. Retinal thickness and vessel density were assessed using the optical coherence tomography angiography. Information on AC was obtained from a comprehensive questionnaire.

**Results:**

A total of 739 children (mean age ± SD: 7.40 ± 0.29 years) had complete eye examination and questionnaire data we needed. Ninety-four children (12.7%) had AC, among which, 5 children had the history of corticosteroid use and were excluded from the final analysis. Spherical equivalent, axial length, body mass index and birth weight were correlated with retinal parameters. After adjusting for sex, age, spherical equivalent, axial length, body mass index, birth weight and premature history, children with AC had thinner retinal nerve fiber layer thickness for average (117.39 versus 120.97 μm, *p* = 0.007), temporal (80.73 versus 84.34 μm, p = 0.001), nasal (98.82 versus 102.18 μm, *p* = 0.049) and inferior (152.68 versus 157.06 μm, *p* = 0.034) quadrants than the control group.

**Conclusions:**

Children with AC tended to have thinner retinal nerve fiber layer thickness. More attention is needed to fundus condition of children with AC.

**Supplementary information:**

The online version contains supplementary material available at 10.1186/s12886-022-02399-7.

## Background

Allergic conjunctivitis (AC), frequently associated with allergic rhinitis and other allergic comorbidities, is the most common form of ophthalmological allergy. Itching is the hallmark of AC, accompanied by redness, watering, swelling, photophobia and blurring of vision. Symptoms may occur in acute episodes, generally recurrent or may persist in a chronic form [1].

The prevalence of AC varies widely from 3.2 to 39.9% in school-aged populations worldwide [2–5]. According to recent research, 28% of school children in metropolitan Shanghai experienced AC symptoms [6]. The International Study of Asthma and Allergies in Childhood (ISAAC) Phase Three study shows an increasing prevalence of allergic rhinoconjunctivitis over time [7], which may increase health care burden. Moreover, AC could reduce patients’ emotional health, social functioning and quality of life, decrease productivity, and could increase costs due to medical treatment [8, 9]. Therefore, AC needs more attention from patients, their parents and ophthalmologists.

A previous study found that AC may induce retinal inflammation, which might promote the progression of myopia in children [10]. So far, few data are available on the relationship between AC and ocular fundus. Whether retinal parameters change in patients with AC remains unknown. In this study, we assessed the association between AC and retinal thickness and microvascular density in a large population-based study of 7-year-old Chinese school-age children in the Nanjing Eye Study (NES).

## Methods

### Study Population

NES is a population-based cohort study in eastern China, aiming to investigate the occurrence and development of ocular diseases in children longitudinally. The details of NES have been reported previously [11, 12]. This study is part of the NES and the data were obtained in 2019, when these children were 7 years old.

The study was approved by the Ethics Committee of the First Affiliated Hospital with Nanjing Medical University and followed the tenets of the Declaration of Helsinki. Written informed consent was obtained from the parents or legal guardians of all children, and oral assent was obtained from all participants right before the examination.

## Eye and Anthropometric Examinations

Visual acuity, stereoacuity test, ocular alignment and motility, refraction, ocular biometric parameters, intraocular pressure (IOP), anterior segment, posterior segment, and optical coherence tomography angiography (OCTA) were conducted on each child, which were performed by a trained team composed of six ophthalmologists and four optometrists.

Noncycloplegic refractive error was performed with the autorefractor (Cannon RF10l; Canon, Tokyo, Japan) and the spherical equivalent (SE) was calculated as the sphere plus half of the cylinder. Axial length (AL) and corneal radius (CR) were performed with IOLMaster-500 (Carl Zeiss Meditec AG, Jena, Germany). IOP was measured using iCare ic 100 (Helsinki, Finland). Anthropometric parameters including height and weight, and body mass index (BMI) was defined as weight (kg)/height (m)^2^.

## Optical coherence tomography angiography

The scanning for thickness and vessel density in retina was performed with OCTA (Optovue RTVue XR Avanti; Optovue, Inc., Fremont, CA, USA). It detected blood flow in an acquired volume using the split-spectrum amplitude decorrelation angiography, an algorithm employed to improve the signal-to-noise ratio by splitting the spectrum to generate multiple repeat OCT frames from the 2 original repeat OCT frames.

The vessel density was automatically measured and defined as the percentage of pixels with flow signal. The macula was imaged with a 6 × 6 mm scan. In this study, the partition of the macula consists of 3 concentric rings: 1 mm center (fovea), 1-3 mm (parafovea), and outer ring of 3-6 mm diameters (perifovea). The optic nerve head was imaged with a 4.5 × 4.5 mm scan and the peripapillary thickness or vessel density was assessed in the 4 mm diameter circle excluding the central 2 mm diameter circle. Superficial vascular complex (SVC), deep vascular complex (DVC) and radial peripapillary capillaries (RPC) were automatically segmented. SVC extended from the internal limiting membrane (ILM) to 10 μm above the lower boundary of inner plexiform layer. DVC extended from 10 μm above the lower boundary of inner plexiform layer to 10 μm below the lower boundary of outer plexiform layer. RPC extended from ILM to the lower boundary of retinal nerve fibre layer (RNFL). Macular thickness (from ILM to RPE) and peripapillary RNFL thickness (from ILM to RNFL) were also measured automatically, so as the area (mm^2^) and perimeter (mm) of the foveal avascular zone (FAZ). Bennett’s formula was used to determine a scaling factor for adjustment for FAZ perimeter and area (scaling factor = 3.46 × 0.013062 × [AL − 1.82]) [13].

Scans with an overall Quality Index ≥ 6 from right eye were included for analysis. Images with obvious decentration, segmentation errors, motion artifacts, defocus, projection artifacts, or stretching defects were excluded from the analysis.

## Questionnaires and definitions

Detailed questionnaires including basic information, birth and feeding history, allergic diseases and daily activities were self-administered to parents or legal guardians of each child to collect information. AC was determined if either of answers to two questions was positive: “Has your child had a problem with recurrent itchy eyes in the past 12 months?” and “Has your child been diagnosed with AC by an ophthalmologist in the past 12 months?” (Supplementary file 1).

There were two questions about the history of eye drop use in the questionnaires: “Has your child used any eye drops?” and “What’ the name of the eye drops that the child has used?”. If the answer to the first question was positive, parents or legal guardians needed to write down the name of the eye drops under the second question, and were encouraged to review medical records and seek help from our certified ophthalmologists if they had any question.

Children with history of any ocular fundus disease, ocular surgery or trauma, unfinished examinations or lack of the questionnaire information were excluded from this study.

### Statistical analysis

Data analysis was performed using the IBM Statistical Package for the Social Sciences statistics (V.13.0, Armonk, NY, USA). *P* value < 0.05 was considered statistically significant. Mean ± standard deviation (SD) was used for continuous measures, and frequency count and percentage were used for the categorical measures. Student’s *t*-test was used to compare means and χ^2^ test was used to compare percentages. Generalized linear models were performed to assess relationship between AC and retinal parameters, and to calculate estimated marginal means of retinal thickness and vessel density in AC group and control group, adjusted for sex, age, SE, AL, BMI, birth weight and premature history. Spearman correlation analysis was used to investigate the associations of retinal parameters with SE, AL, BMI and birth weight.

## Results

A total of 739 children (mean age ± SD: 7.40 ± 0.29 years) had complete eye examination and questionnaire data. Ninety-four children (12.7%) had AC, among which, 5 children had the history of corticosteroid use and were excluded from the following analysis since long-term corticosteroid use may affect RNFL thickness [14]. Finally, 89 children with AC and 645 controls without AC were included in the final analysis. Comparisons of characteristics between AC group and control group without AC were shown in Table 1. There were no significant differences in age, sex, height, weight, BMI, birth weight, preterm history, SE, AL, AL/CR and IOP between the two groups (all *p* ≥ 0.05).

**Table 1:** Comparisons of characteristics between AC group and control group

Parameters^a^	Control (N = 645)	AC (N = 89)	p value^b^
Demographics			
Age, m	88.80(3.52)	88.37(3.19)	0.280
Sex (Boys), %	330(51.2%)	48(53.9%)	0.624
Anthropometry			
Height, cm	128.99(6.44)	128.97(6.36)	0.988
Weight, kg	27.67(5.88)	27.44(6.12)	0.724
BMI, kg/m2	16.52(2.62)	16.33(2.43)	0.515
Birth parameters			
Birth weight, g	3.30(0.52)	3.26(0.44)	0.550
Preterm history, %	37(5.7%)	7(7.9%)	0.428
Ocular parameters			
SE, D	-0.32(0.80)	-0.27(0.67)	0.581
AL, mm	22.98(0.77)	23.02(0.79)	0.695
AL/CR	2.95(0.08)	2.96(0.09)	0.317
IOP, mmHg	18.56(2.47)	18.77(2.40)	0.461

^a^ Presented as mean (SD) or number (%)

^b^*P* values for the difference between AC group and control group were assessed by *t-*test for means or χ^2^ test for percentages

Abbreviations: *AC* allergic conjunctivitis; *N* number; *BMI* body mass index; *SE* spherical equivalent; *D* diopter; *AL* axial length; *CR* corneal radius; *IOP* Intraocular pressure

Table 2 showed the Spearman correlation for the association of retinal thickness and vessel density with SE, AL, BMI and birth weight. We found SE was positively correlated with average macular thickness (Spearman’s correlation coefficients *r* = 0.097, *p* = 0.008), average RNFL thickness (*r* = 0.105, *p* = 0.005), but was negatively correlated with foveal thickness and foveal SVC density (*r* = -0.083, *p* = 0.025). AL was negatively correlated with average macular thickness (*r* = -0.231, *p* < 0.001), average RNFL thickness (*r* = -0.123, *p* = 0.001), perifoveal SVC density (*r* = -0.084, *p* = 0.027) and average DVC density (*r* = -0.087, *p* = 0.023), but was positively correlated with foveal thickness, foveal SVC density and foveal DVC density (*p* < 0.001). BMI was positively correlated with foveal thickness (*r* = 0.122, *p* = 0.001), foveal SVC density (*r* = 0.073, *p* = 0.049), foveal DVC density (*r* = 0.077, *p* = 0.036) and RPC density (whole image: *r* = 0.082, *p* = 0.025; peripapillary: *r* = 0.093, *p* = 0.011). However, BMI was negatively correlated with FAZ area (*r* = -0.086, *p* = 0.023) and FAZ perimeter (*r* = -0.083, p = 0.030). As for birth weight, parafoveal thickness (*r* = 0.081, *p* = 0.028), average RNFL thickness (*r* = 0.126, *p* = 0.001) and RPC density (peripapillary: *r* = 0.098, *p* = 0.008) had positive correlation with birth weight.

**Table 2:** Spearman correlation for associations of retinal parameters with spherical equivalent, axial length, body mass index and birth weight

	Spherical equivalent		Axial length		Body mass index		Birth weight	
	Correlation coefficient	*p* value	Correlation coefficient	*p* value	Correlation coefficient	*p* value	Correlation coefficient	*p* value
Macular Vessel Density								
SVC density, %								
Average	0.033	0.374	-0.059	0.121	-0.037	0.314	0.006	0.880
Fovea	***-0.083***	***0.025***	***0.193***	***< 0.001***	***0.073***	***0.049***	0.059	0.111
Parafovea	0.010	0.778	-0.032	0.399	-0.041	0.270	-0.012	0.748
Perifovea	0.049	0.184	***-0.084***	***0.027***	-0.043	0.243	0.006	0.880
DVC density, %								
Average	0.038	0.303	***-0.087***	***0.023***	-0.055	0.139	-0.019	0.606
Fovea	-0.071	0.055	***0.212***	***< 0.001***	***0.077***	***0.036***	0.043	0.241
Parafovea	0.020	0.589	-0.072	0.059	-0.048	0.190	-0.013	0.731
Perifovea	0.043	0.244	***-0.099***	***0.009***	-0.060	0.105	-0.022	0.548
FAZ								
Area, mm2	0.040	0.291	-0.022	0.570	***-0.086***	***0.023***	0.020	0.596
Perimeter, mm	0.033	0.384	-0.029	0.443	***-0.083***	***0.030***	0.015	0.695
Macular thickness, µm								
Average	***0.097***	***0.008***	***-0.231***	***< 0.001***	-0.018	0.625	0.060	0.105
Fovea	***-0.083***	***0.025***	***0.140***	***< 0.001***	***0.122***	***0.001***	0.043	0.242
Parafovea	0.035	0.343	***-0.077***	***0.043***	0.027	0.471	***0.081***	***0.028***
Perifovea	***0.120***	***0.001***	***-0.276***	***< 0.001***	-0.036	0.324	0.050	0.175
Peripapillary RNFL thickness								
Average	***0.105***	***0.005***	***-0.123***	***0.001***	0.021	0.561	***0.126***	***0.001***
Temporal	0.033	0.366	0.036	0.349	-0.011	0.764	***0.117***	***0.002***
Superior	0.060	0.104	***-0.110***	***0.004***	-0.014	0.701	***0.092***	***0.013***
Nasal	***0.110***	***0.003***	***-0.105***	***0.006***	0.044	0.230	***0.084***	***0.024***
Inferior	***0.096***	***0.009***	***-0.165***	***< 0.001***	-0.006	0.864	***0.098***	***0.008***
RPC density, %								
Whole Image	-0.002	0.953	0.004	0.911	***0.082***	***0.025***	0.048	0.194
Peripapillary	0.007	0.848	0.031	0.416	***0.093***	***0.011***	***0.098***	***0.008***

Abbreviations: *SVC* superficial vascular complex; *DVC* deep vascular complex; *FAZ* foveal avascular zone; *RNFL* retinal nerve fibre layer; *RPC* radial peripapillary capillaries

Bold and italic font indicates statistical significant

Table 3 showed retinal thickness and vessel density between AC group and control group that adjusted for sex, age, SE, AL, BMI, birth weight and premature history. There were no significant differences in SVC density, DVC density, FAZ or RPC density between AC and control groups after adjusting for confounding factors (*p* ≥ 0.05). There was no difference in macular thickness between two groups (*p* ≥ 0.05). Differently, the average RNFL thickness in the AC eyes, 117.39 ± 11.31 μm, was thinner compared to the control eyes, 120.97 ± 11.29 μm, with a difference of -3.584 μm (*p* = 0.007). RNFL thickness for temporal (80.73 versus 84.34 μm, *p* = 0.001), nasal (98.82 versus 102.18 μm, *p* = 0.049) and inferior (152.68 versus 157.06 μm, *p* = 0.034) quadrants followed a similar pattern for AC and control groups.

**Table 3:** Multivariate analysis for the association of AC with retinal thickness and vessel densitya

	Control	AC	Mean difference	95% CI for mean difference	*p* value
	Mean value^b^	Mean value^b^			
Macular Vessel Density					
SVC density, %					
Average	50.38(2.96)	50.56(2.97)	0.178	(-0.502,0.858)	0.608
Fovea	21.49(6.64)	21.80(6.65)	0.315	(-1.208,1.838)	0.685
Parafovea	52.11(4.13)	52.63(4.14)	0.524	(-0.424,1.472)	0.278
Perifovea	50.95(2.84)	51.01(2.84)	0.069	(-0.582,0.720)	0.836
DVC density, %					
Average	52.97(4.65)	52.52(4.65)	-0.445	(-1.510,0.620)	0.412
Fovea	35.73(7.24)	35.92(7.25)	0.187	(-1.473,1.846)	0.825
Parafovea	56.76(3.54)	56.43(3.54)	-0.327	(-1.138,0.484)	0.429
Perifovea	52.49(5.22)	51.98(5.23)	-0.503	(-1.700,0.694)	0.409
FAZ	0.00(0.00)				
Area, mm^2^	0.27(0.09)	0.27(0.09)	-0.002	(-0.023,0.019)	0.869
Perimeter, mm	1.97(0.36)	1.97(0.36)	-0.001	(-0.082,0.081)	0.988
Macular thickness, µm					
Average	291.76(10.84)	293.33(10.86)	1.567	(-0.919,4.052)	0.216
Fovea	236.92(16.49)	237.65(16.52)	0.731	(-3.052,4.513)	0.705
Parafovea	318.42(12.18)	319.67(12.20)	1.243	(-1.551,4.037)	0.383
Perifovea	285.86(11.20)	287.56(11.22)	1.702	(-0.867,4.271)	0.194
Peripapillary RNFL thickness					
Average	***120.97(11.29)***	***117.39(11.31)***	***-3.584***	***(-6.174,-0.995)***	***0.007***
Temporal	***84.34(9.46)***	***80.73(9.47)***	***-3.611***	***(-5.781,-1.442)***	***0.001***
Superior	147.10(16.23)	144.54(16.25)	-2.561	(-6.283,1.160)	0.177
Nasal	***102.18(14.56)***	***98.82(14.58)***	***-3.356***	***(-6.695,-0.017)***	***0.049***
Inferior	***157.06(17.68)***	***152.68(17.70)***	***-4.376***	***(-8.430,-0.323)***	***0.034***
RPC density, %					
Whole Image	49.99(1.92)	49.70(1.93)	-0.295	(-0.736,0.147)	0.190
Peripapillary	52.28(2.43)	52.05(2.43)	-0.231	(-0.788,0.325)	0.415

^a^ Generalized linear models were performed to assess association between AC and retinal parameters, and to calculate estimated marginal means of retinal thickness and vessel density in AC group and control group, adjusted for sex, age, spherical equivalent, axial length, body mass index, birth weight and premature history

^b^ Presented as mean (SD)

Abbreviations: *AC* allergic conjunctivitis; *CI* confidence interval; *SVC* superficial vascular complex; *DVC* deep vascular complex; *FAZ* foveal avascular zone; *RNFL* retinal nerve fibre layer; *RPC* radial peripapillary capillaries

Bold and italic font indicates statistical significant

## Discussion

AC is known as an immunological inflammatory process of the ocular anterior surface, whereas the impact of AC on the retinal microvasculature and thickness has not been clarified. In this study, we found that children with AC tended to have thinner RNFL thickness compared to controls with a difference of about 4 μm. According to previous studies about the RNFL thickness change detected by OCT in patients with glaucoma, a short-term decrease in average RNFL thickness of 4–5 μm may be considered to have progression of glaucoma [15, 16]. This finding suggests that a decrease of 4 μm in the RNFL thickness might have adverse impact in vision. In addition, considering that the average RNFL thickness in children without AC is approximately 120 μm in our study and the minimum of the RNFL thickness reported by literature is approximately 50 μm, a difference of 4 μm represents 5.7% of the range from 120 μm to 50 μm, which might be more than a tiny change. Base on the above reasons, we think that the difference of 4 μm in the RNFL thickness between children with AC and controls is not just statistically significant, but also clinically relevant.

Immunoglobulin-E (IgE) mediated mast cell degranulation and/or T-lymphocyte-mediated immune response is the immune mechanism of AC [17]. Allergens include pollen, animal dander and other airborne antigens cause cross-linkage of membrane-bound IgE and trigger mast cells to degranulate within a few minutes of allergen stimulation, releasing a range of allergic and inflammatory mediators, such as histamines, proteases and leukotrienes [18]. Histologically, AC is characterized by the conjunctival infiltration of inflammatory cells, including neutrophils, eosinophils, lymphocytes, and macrophages [10].

In an animal experiment, tumor necrosis factor α, interleukin-6 (IL-6), IL-8, and chemoattractant protein-1 in AC retinas were higher while IL-10 was lower compared to the control group [10], and we used OCTA to confirm that AC did have an effect on retina, indicating that AC is not only an immunological inflammatory process of the ocular anterior surface, but also associated with ocular fundus. In allergic rats’ sclera and retinas, the expression of transforming growth factor β and matrix metalloproteinase-2 (MMP2) were higher and expression of collagen type I (COL-I) was lower [10]. MMP2 is an enzyme that breaks down COL-I, while COL-I is the main extracellular matrix in the sclera and its degradation will result in thinning of the sclera. Thus, it is reasonable to assume that it may be the degradation of certain molecules in RNFL that eventually result in its thinning. And considering the recidivity of AC, the higher level of inflammatory factors could affect retina continuously. Another reason might be that chronic inflammation caused by AC induce oxidative stress, an imbalance between overproduction of oxidant compounds and inadequate anti-oxidant defense, resulting in axonal nerves loss and ganglion cells death [19, 20]. More experiments are needed to support our hypothesis.

In this study, no association was found between AC and retinal vessel density. On the one hand, the central retinal artery and short posterior ciliary artery that branch from the ophthalmic artery supply the retina and choroid, and the conjunctiva is fed by anterior ciliary arteries and other arteries, branches of the ophthalmic artery [21]. Since retina and conjunctiva are supplied by different branches of the ophthalmic artery, the retinal blood flow may not be significantly affected by conjunctival blood flow. On the other hand, since information on AC was obtained through questionnaires, it is unclear whether the AC is active, which may affect our negative findings on retinal vessel density. Hence, whether active and inactive AC have different effects on retinal vessel density requires further research. What’s more, whether the retinal blood flow changes as the disease progresses remains unclear and needs long-term follow-up.

Our study and other previous studies suggest that AC patients may be at higher risk for impairment in retinal structure, dry eye, myopia, and keratopathy (corneal ulcers, pannus, keratoconus, etc.) [10, 22–24]. Therefore, ophthalmologists may need to monitor AC patients for the changes in refraction, ocular surface, fundus and other ocular diseases.

In the present study, we found average macular thickness and average RNFL thickness were thicker with increasing SE, while foveal thickness and foveal SVC density were decreased with increasing SE. In regard to the relationship between SE and foveal parameters, different studies had different results and some studies found no association between SE and foveal parameters or foveal thickness was positively correlated with SE [25, 26]. In other studies, foveal thickness was negatively correlated with SE, which is consistent with our results [11, 27]. One possible mechanism is that peripheral retinal thickness is reduced in order to preserve the thickness of the central retina, which is essential for visual function [28]. Our study found that AL was negatively correlated with average macular thickness, average DVC density, perifoveal SVC density and average RNFL thickness, while positively correlated with foveal thickness and foveal vessel density, and similar findings have been reported in other researches [11, 25, 27, 29–31]. While some other studies found AL was positively correlated with SVC density and negatively correlated with RPC density [32, 33]. Obese children tend to have higher values of foveal thickness, foveal SVC and DVC densities [34, 35], comparing favorably with our study. Our study also found BMI was positively correlated with RPC density, negatively correlated with FAZ and had no association with RNFL thickness, in accordance with findings by Zhu et al. that RNFL thickness had no significant correlation with BMI [30]. Some other studies found morbid obesity group had lower RNFL thickness or BMI did not influence the RPC or FAZ [34, 36, 37]. Cheung et al. found BMI was negatively correlated with FAZ area in univariable analysis, but it was no longer significant in multiple analysis [32]. Low birth weight and prematurity were found to be associated with thickening of the fovea, thinner parafoveal thickness and thinner RNFL thickness [11, 38–40]. In our research, birth weight was positively correlated with parafoveal thickness, RNFL thickness and RPC density.

To our knowledge, this is the first report that used OCTA to explore the ocular fundus of patients with AC. What’s more, it is a large population-based study, making the findings more credible. We acknowledge some limitations in this study. Firstly, the diagnosis of AC was obtained through questionnaires, which may cause recall bias. And whether the AC is active can’t be obtained. Secondly, due to the OCTA mode we chose, the macula can’t be analyzed for each layer in detail. Thirdly, non-cycloplegic refraction data were used in our study, which might have potential effect on the results. Fourthly, the long-term changes of retinal microstructure and microvasculature in AC children could not be determined in this cross-sectional study. Data from longitudinal follow-ups, other regions, and age groups are needed.

## Conclusions

In conclusion, in this large population-based study of Chinese school children aged 7 years, we found 12.7% children had AC. Children with AC tended to have thinner RNFL thickness. Fundus condition of children with AC needs more attention.

## Supplementary information

**Additional file 1.** Questionnaire; The English version of the questionnaire used in this study.

## Data Availability

All data included in this study are available from the corresponding author upon reasonable request.

## Abbreviations

AC: Allergic conjunctivitis
ISAAC: The International Study of Asthma and Allergies in Childhood
NES: the Nanjing Eye Study
IOP: intraocular pressure
OCTA: optical coherence tomography angiography
SE: spherical equivalent
AL: axial length
CR: corneal radius
BMI: body mass index
SVC: superficial vascular complex
DVC: deep vascular complex
RPC: radial peripapillary capillary
ILM: internal limiting membrane
RNFL: retinal nerve fibre layer
FAZ: foveal avascular zone
SD: standard deviation
IgE: Immunoglobulin-E
IL: interleukin
MMP2: matrix metalloproteinase-2
COL-I: collagen type I
