# Supplementary material for: Altered retinal nerve fiber layer thickness in children with allergic conjunctivitis: the Nanjing eye study
Source: BMC Ophthalmol. 2022 Apr 22;22:183. doi: 10.1186/s12886-022-02399-7 (PMC9028092; doi:10.1186/s12886-022-02399-7)
Supplement: Supplementary file 1 — Additional file 1. Questionnaire; The English version of the questionnaire used in this study. [file 12886_2022_2399_MOESM1_ESM.pdf]

Questionnaire: The English version of the questionnaire used in this study.

School Class Name Sex: Male Female Date of birth

1.Gestational age

☐<37 weeks ☐≥37, <42 weeks ☐≥42 weeks

2.Birth weight: _____kg

3.Has your child had a problem with recurrent itchy eyes in the past 12 months?

☐Yes ☐No

4.Has your child been diagnosed with AC by an ophthalmologist in the past 12 months?

☐Yes ☐No

5.Has your child used any eye drops?

☐Yes ☐No

6.What’s the name of the eye drops that the child has used?

____
